# The Road Less Travelled: A Micro-Costing Analysis of an Online Pre-Death Grief and Loss Programme for Carers of People with a Rare Dementia

**DOI:** 10.1177/00469580251332770

**Published:** 2025-04-21

**Authors:** Bethany F. Anthony, Jill Walton, Emily V. Brotherhood, Sebastian J. Crutch, Rhiannon T. Edwards

**Affiliations:** 1 Bangor University, UK; 2 University College London (UCL), UK

**Keywords:** rare dementia, dementia carers, grief and loss, micro-costing, budget impact

## Abstract

The economic contribution of family and friend carers of people with dementia is substantial. Pre-death grief experienced by carers of people living with a rare dementia is complex as carers are faced with unique challenges due to geographical isolation and a lack of access to shared experiences. There is an urgent need for specialised interventions to support carers lacking local support. A micro-costing analysis of a novel online group-based pre-death grief and loss programme (‘The Road Less Travelled’) tailored for the carers of people with rare dementias was conducted from a provider perspective. Sensitivity analysis was conducted to explore the potential costs of face-to-face delivery of the programme. A budget impact analysis was also conducted to explore the potential costs of roll-out of the programme to carers of people living with a rare dementia across the UK. The total cost of delivering three waves of the grief and loss programme to a total of 20 participant carers was £9848, which equates to a cost of £492 per carer participant. Sensitivity analysis indicated a total cost of £14 673 for face-to-face delivery, equating to £734 per carer participant. Sensitivity analysis indicated a total cost of £14 673 for face-to-face delivery, equating to £734 per carer participant. We estimate from our budget impact analysis that the total costs of a UK wide roll-out to people living with a rare dementia (based on 5% of all people with a dementia) would be £21.77 million. To our knowledge, this is first costing analysis of a pre-death grief and loss programme for carers of people living with a rare dementia. These initial assessments of costs provide a base case for future costing analyses and full economic evaluations which can assess both the cost and benefits to society from supporting people with rare dementias and their carers.

**What do we already know about this topic?**

The total cost of dementia care in the UK was £34.7 billion in 2019 and is expected to reach £94.1 billion by 2040. Unpaid care from family and friends provides a significant economic contribution. Pre-death grief is common among carers and is linked to increased depression and caregiver burden.

**How does your research contribute to the field?**

This study found that the cost of delivering the grief and loss programme was £492 per carer participant. We estimate from our budget impact analysis that the total costs of a UK wide roll-out to people living with a rare dementia (based on 5% of all people with a dementia) would be £21.77 million.

**What are your research’s implications towards theory, practice, or policy?**

This is the first cost analysis of a pre-death grief programme for carers of people living with rare dementias. These initial cost estimates can be used to inform future economic evaluations of the programme’s societal costs and benefits.

## Introduction

Approximately 885 000 people have dementia in the UK and of these, it is estimated that between 5% and 15% have a rare dementia typically occurring before the age of 65.^1,2^ In 2019, the total cost of dementia care in the UK was £34.7 billion and this is expected to increase to £94.1 billion by 2040. In 2019, unpaid care accounted for 40% of the total cost of dementia care.^2^ Due to the significant financial contribution of carers of people with dementia and the negative impacts on health and wellbeing associated with their caring role, the World Health Organisation (WHO) global action plan on the public health response to dementia 2017-2025 highlighted the need to support dementia carers through accessible evidence-based information, training programmes and support that is tailored to the needs of carers to help them manage and prevent stress and health issues.^3^

Family and friend carers of people with dementia are often referred to as the invisible second patient as their role is known to increase their risk of psychological distress and adverse health outcomes.^4^ According to Singer and colleagues, pre-death grief is an umbrella term used to describe grief that takes place before the death of a person with a life-limiting illness, and can be further separated into two constructs: anticipatory grief and illness-related grief. Anticipatory grief has been conceptualised as future-focused grief where the grief experienced is focused on the anticipatory losses that will take place after the death of a loved one. Illness-focused grief focusses on the present and is concerned will ongoing losses experienced during the illness as they cope with changes to their own identity and role.^5^ The concept of pre-death grief is highly prevalent among carers of people living with dementia and is associated with depressive symptoms, carer burden and prolonged post-death grief.^6-8^ Pre-death grief among carers of people living with dementia is a multi-faceted reaction to past, present and future losses while caring for their loved one and is shaped by cultural and social factors.^9^ A systematic review reported that 10-38% of carers of people living with dementia experience a high level of pre-death grief.^10^

Pre-death grief can impact individuals over many years and is a particularly complex process among those caring for people with rare dementia which frequently occur earlier on in life before the age of 65. Results of a 2019 study assessing survival and life-expectancy in a cohort of people with early onset dementia revealed that the mean survival time after the onset of symptoms was 209 months (95% CI [185-233]) and 120 months after diagnosis (95% CI [110-130]).^11^ Moreover, compared to carers of people with more typical forms of dementia, who may be supported by established networks and more predictable disease progression, carers of people with rare forms of dementia are faced with unique challenges due to uncertain diagnostic pathways and limited access to tailored support.^12,13^ A diagnosis of young onset dementia can deeply affect family dynamics including issues around childcare responsibilities and financial difficulties due to loss of employment.^14^ The development of targeted interventions is therefore crucial to support the unique complexities of pre-death grief in this population. Previous research has assessed interventions for pre-death grief among dementia carers but have predominantly focused on typical forms of dementia,^15-17^ and therefore may not be suitable to support the complex process of pre-death grief among rare dementia carers. The National Institute for Health and Care Excellence (NICE) recognises the unique challenges of rare conditions, often involving high costs drugs, and have developed specific guidance and frameworks to account for the special nature of rare diseases.^18^ The purpose of the present study was to assess the delivery costs of The Road Less Travelled which is an on online pre-death grief and loss programme for carers of people with a rare dementia. The programme was informed by literature searches that identified a lack of evidence for pre-death grief and loss interventions for carers of people living with rare dementia. The Road Less Travelled programme’s theoretical foundation draws from established concepts from the dementia pre-death grief and loss literature.^19-24^ A flow diagram depicting the programme development and design is available in Supplemental Appendix A. The Road Less Travelled programme formed part of the larger Rare Dementia Support (RDS) Impact study of multicomponent support groups for people with, or supporting someone with, a rare form of dementia.^25^ The term rare dementia is used to describe dementias that affect a relatively small number of people compared to more common forms such as Alzheimer’s disease.^26^ The RDS service, led by the Dementia Research Centre at University College London, supports a membership of 7523 individuals with, working with, or living alongside a rare dementia. Accurate prevalence statistics for rare dementias are unavailable due to challenges of diagnosis, but it is estimated that rare forms of dementia (atypical, vascular or inherited) affect approximately 25% of people living with dementia with many of these occurring before the age of 65.^27^ Other sources give a prevalence statistic of between 5% and 15% of all cases of dementia.^1^ The RDS service offers support for low prevalence and young onset forms of dementia including: frontotemporal dementia (FTD), primary progressive aphasia (PPA), posterior cortical atrophy (PCA); directly inherited conditions [familial Alzheimer’s disease (FAD), familial FTD (fFTD)] and Lewy Body Dementia (LBD) and Young Onset Alzheimer’s Disease (YOAD). Support comprises multicomponent offers such as: in-person and online large group seminars/webinars,1:1 online support, monthly peer to peer facilitated online discussions, and themed online support programmes. To our knowledge, this is the first costing analysis of a pre-death grief interventions for carers of people living with a rare dementia.

## Methods

From a provider perspective, this study undertook a micro-costing approach to cost the delivery of one such RDS online support programme, ‘The Road Less Travelled’. This programme was designed with and for rare dementia carers, tailored to supporting those facing bereavement in the future, and who may be experiencing grief while caring for a loved one who is physically present while psychologically changing, where there is no hope of a reconciliation due to the insidious nature of the dementia syndrome.^28^ The programme was specifically developed to support family or friend carers of a person with a rare dementia, to allow them to explore their feelings of pre-death grief and loss, share experiences with others, and consider future plans. The reporting of this study followed the Consolidated Health Economic Evaluation Reporting Standards 2022 (CHEERS 2022) guidelines^29^ (see Supplemental Appendix B). As the programme formed part of the larger RDS Impact study of multicomponent support groups, a formal health economics analysis plan for this component of work was not pre-specified.

The programme was delivered online by two facilitators with professional and personal experiences of rare dementia to small groups of carer participants located in different regions across the UK. The micro-costing analysis presented in this paper costed the delivery of three waves of the programme delivered between 9th February 2021 and 12th January 2022. The programme was delivered online and was facilitated from London to RDS members across the UK. Each wave typically consisted of six sessions; however, an optional seventh session was offered if required, to allow facilitators to cover the material adequately, providing for a more participant focused approach to the delivery and allowing for additional discussion time if a particular issue warranted it amongst participants. This analysis included three waves of the programme; two of the waves consisted of six 2-hour sessions (waves 1 and 3), and one wave consisted of seven 2-hour sessions (wave 2). Sessions delivered in each of the waves were delivered on a fortnightly basis and each wave of the programme took approximately 3 months to deliver. The total number of carer participants in the programme was 20: nine in wave 1, four in wave 2, and seven in wave 3.

### Base Case Analysis

From a provider perspective, we conducted a micro-costing analysis to explore the direct costs of programme delivery including staff time and material costs.^30^ The provider perspective included all upfront costs borne by the university research centre. A bottom-up micro-costing approach was used as it is a particularly useful method of measuring the economic costs of new interventions through the collection of detailed resource use data which allows accurate costing of a programme.^31,32^ The two programme facilitators completed cost diaries for each of the three waves (Supplemental Appendix C). Costs collected in the diaries included time spent delivering the sessions, activities completed in preparation for the sessions (eg, session planning and scheduling) and post-session activities such as catch-ups and debriefs. Facilitator time was costed using their hourly research facilitator role salary rate including on-costs. Material costs were collected which consisted of printing and postage costs (including Value-Added Tax) of handbooks sent to carer participants and members of the RDS team. The unit cost of the handbooks varied across waves as handbooks were updated/revised from wave to wave and a different printing service was used for one wave. The total costs of materials included the cost of copies provided to facilitators, RDS team members (eg, co-ordination) and also any participants who initially accepted to take part in the programme but subsequently did not continue their participation. Costs were presented in British Pounds Sterling (£) for the cost year 2022. In accordance with National Institute for Health and Care Excellence (NICE) guidelines, we did not apply a discount rate as the time horizon of the study was less than 12 months.^29,33^

Our base case analysis explored the upfront costs of delivering the programme across three waves of the programme (delivered to 20 carer participants in total) in an online format and produced descriptive statistics including the total costs of three waves of delivery and an estimated mean cost per carer participant. Training costs were not included in our calculations.

### Sensitivity Analysis

In order to explore how different overhead costs could impact the programme costs, sensitivity analysis was conducted to vary the resources, to include a range percentage uplifts for overheads (valued at 25%, 50%, 75% and 100% of total facilitator costs). These scenarios were illustrative to explore the programmes sensitivity to moderate and substantial cost changes, enabling the evaluation of the programmes vulnerability to cost fluctuations to inform decision making going forward. In addition, as part of a deterministic sensitivity analysis, an alternative delivery scenario was modelled to estimate the potential costs of delivery in a face-to-face format compared with online delivery mode (utilised over the Covid-19 pandemic) using cost estimates provided by the RDS study team including venue hire and catering costs.

Based on published estimates on the number of people with a rare dementia in the UK, we conducted a budget impact analysis (BIA) to explore the potential costs of roll-out of the programme across the UK. BIAs are economic assessments used to explore the financial consequences of implementing a new intervention and are used to support decision making regarding the allocation and reallocation of resources within specific settings with finite resources.^34,35^

## Results

### Base Case Analysis

The total cost of delivering the grief and loss programme across the three waves to 20 participant carers was £9848. This equates to £492 per carer participant. Total costs of programme delivery comprised of facilitator costs and materials (Table 1). The total cost of facilitator activities was £9123; the largest driver of cost was time spent planning the sessions, followed by the time spent delivering the sessions (Table 1). The total time spent delivering sessions and completing pre- and post-session activities was 320.5 hours. The total cost of materials was £724 (including VAT), which consisted of the cost of printing the handbooks and any postage costs incurred (Table 1). There was also a separate set-up cost of £43 for a user licence that was not included in our base case analysis as this cost did not directly fall under the delivery costs of the three waves included in our analysis.

**Table 1.** Total Time and Costs of Programme Delivery Across Three Waves (Time Accumulated by Two Facilitators and Materials).

Facilitator activities	Time (hours)	Costs (£)
Meeting to co-ordinate sessions and themes with RDS team	8	£221.00
Planning the sessions (individually and with the co-facilitator)	85	£2419.30
Finalising dates and details with co-ordinator	10	£276.25
Writing descriptions of groups and other course material, slides, etc.	30	£848.16
Inviting or liaising with members ahead of first session	18	£555.48
Delivering sessions	76	£2099.50
Immediate post-session discussions and debriefs with co-facilitators	25	£690.63
Facilitator discussion groups	12	£331.50
Liaising with and supporting group members one-to-one between sessions	56.5	£1682.13
Total facilitator costs	320.5	£9123.94
Materials (printing and postage of handbooks [inc. VAT])	NA	£724.49
Total programme cost	NA	£9848.43

### Sensitivity Analysis Varying Overheads

We conducted sensitivity analysis to account for overheads that were not included in our base case analysis. Overheads were valued at 25%, 50%, 75% and 100% of total facilitator costs equating to £2281, £4562, £6843 and £9124, respectively. Based on a 25% overhead uplift on facilitator costs, the total cost of the programme would be £12 129 (Table 2). A 100% overhead uplift would result in a total programme delivery cost of £18 972 (Table 2).

**Table 2.** Total Costs of Programme Delivery Accounting for Overheads.

Scenario	Total costs
Base case (no overheads)	£9848.43
25% overhead uplift	£12 129.41
50% overhead uplift	£14 410.40
75% overhead uplift	£16 691.38
100% overhead uplift	£18 972.37

### Scenario Sensitivity Analysis to Estimate the Costs of Face-to-Face Delivery

Sensitivity analysis was conducted to estimate the potential costs of face-to-face delivery of the programme based on the calculations from our base case analysis and additional venue hire and catering cost estimates provided by the RDS team. Estimated total costs for face-to-face delivery of the programme was £14 673 based on the total costs accrued in our base case analysis (facilitator costs and materials) and additional venue hire and catering costs (Table 3). This equates to £734 per carer participant. Venue hire was valued at £4750 at a cost of £250 per session for the 19 sessions delivered across the three waves. Estimated catering costs were £75 to account for light refreshments provided to the 20 carers that attended the programme in total across the three waves, calculated at £3.75 per carer participant (Table 3).

**Table 3.** Scenario Sensitivity Analysis for Face-to-Face Delivery.

Cost component	Total cost
Facilitator costs	£9123.94
Materials (printing and postage of handbooks [inc. VAT])	£724.49
Room hire (£250 per session)	£4750.00
Catering (£3.75 per person)	£75.00
Total	£14 673.43

### Budget Impact Analysis

Based on published estimates on the number of people with a rare dementia in the UK, we conducted a budget impact analysis (BIA) to explore the potential costs of roll-out of the programme across the UK. Our base case analysis estimated that the cost per carer participant to partake in the grief and loss programme was £492. According to a report commissioned by the Alzheimer’s Society, the number of people with dementia in the UK is estimated at 885 000.^2^ It is estimated that between 5% and 15% of people with dementia have a rare type of dementia which frequently occur earlier on in life before the age of 65.^1,36^ This equates to between 44 250 and 132 750 people with a rare dementia in the UK. Using the cost per carer participant calculated in our base case analysis and these estimates of the number of people with a rare dementia, total costs of roll-out of the programme to one carer for every person with a rare dementia in the UK would be between £21.77 million and £65.31 million. Given that estimates of rare dementia prevalence are scarce and largely based on the experience of specialist centres, it is reasonable to assume that only a small proportion of those with a rare dementia will ever receive the diagnosis they deserve, and so it may be appropriate to consider the lower end of the 5-15% prevalence estimate range when taking into account these projected costs.

## Discussion

The total costs of delivering three waves of The Road Less Travelled programme to 20 carer participants was £9848 at an estimated cost per carer participant of £492. Sensitivity analysis revealed that if the programme were to be delivered face-to-face to the same extent (ie, number of participants, sessions etc.), the total cost of programme delivery would increase to £14 673 (£734 per participant) to account for venue hire and catering costs. Based on the estimated cost per participant of £492 from the base case analysis and prevalence statistics of rare dementia,^36^ the potential budget impact if the online programme were to be rolled-out to one carer for every person with a rare dementia in the UK would be between £21.77 million and £65.31 million.

This study used robust micro-costing and BIA methods to estimate the costs of a novel online pre-death grief and loss programme for carers of people with rare dementia for the first time. The sensitivity analysis which projected the additional costs for face-to-face delivery were estimated and may undervalue the actual costs of in-person delivery. For example, we did not consider additional administration costs (owing to The Road Less Travelled being set up in conjunction with other simultaneously occurring small themed groups, thus separable administration costs were not obtainable). Variation in costs will also differ depending on how far away the person living with dementia lives and also whether they utilise paid carers. We did not consider facilitator travel time or whether the number of participants would likely vary given the geographical area that the groups were held in. In addition, there may be other potential variations for face-to-face delivery such as the length of session, the number of facilitators and the location. Therefore, there is uncertainty around how these costs may differ, particularly across geographical locations where costs may be higher due to premium costs for venue hire and more demand on public spaces (eg, in London). Further analysis should look to collect data on the resources required to deliver in-person sessions and estimate the costs of delivery across a range of potential venue types which could be utilised to host these sessions ranging from high-cost private venues to low-cost hospital, charity or community venues.

The study perspective adopted was a narrow provider perspective and did not extend to consider indirect costs such as productivity losses for the participants’ time attending the programme and any potential travel or respite care costs, which are important when considering the opportunity cost of delivering the programme. Given the human costs of dementia and the costs to health and social care, future research may appropriately adopt a wider societal perspective perhaps using a social cost benefit analysis approach as advocated by the Treasury which captures a full range of costs and benefits from roll-out of this programme.^37^ Moreover, when considering future programme rollout, it also must be noted that the cost of programme delivery will vary significantly depending on the facilitators’ credentials. Although dementia support workers were employed in this study, other professionals such as nurses, speech and language therapists and palliative care specialists could be used to deliver the programme within the NHS. The key requirement is an appreciation of rare dementia diagnoses and their impact on the experience of caregiver grief, rather than a specific professional background per se.

The costs of grief and loss programmes for carers have not been established previously in the literature, limiting our ability to compare our findings with published evidence. Previous studies have reported on pre-death grief programmes for dementia carers and carers of adults with a life-threatening illness, but none of these studies explored costs.^16,38,39^ There is also limited evidence on the costs and cost-effectiveness of online interventions for dementia carers in general.^40^ A recent study published by Henderson and colleagues assessed the cost-effectiveness of online cognitive behavioural therapy (CBT) for dementia carers experiencing anxiety and depression. The economic evaluation took a health and social care perspective and compared online CBT with or without telephone support with a standard psychoeducation care treatment. Total costs at 26 weeks were £358, £458 and £458 for the psychoeducation, online CBT (without telephone support) and online CBT (with telephone support), respectively. Results found that the online CBT (with telephone support) produced 0.003 greater quality-adjusted life years (QALY) compared with the psychoeducation intervention. Moreover, online CBT (without telephone support) produced a greater QALY gain compared with the online CBT intervention (with telephone support).^40^

While it is acknowledged that CBT is a well-established intervention, this novel pre-death grief and loss programme offers a unique approach and there are currently no directly comparable programmes. Notably, this analysis has reported a lower hourly cost per participant than individual CBT, which typically costs between £60 and £100 per hour in the UK.^41^ The Road Less Travelled is a centralised programme that provides specialised knowledge and consistent support, reaching carers across a wide geographical area and fostering a sense of community among those with shared experiences. This specialised support is crucial for individuals facing the unique challenges of rare dementias, often lacking adequate local resources. Due to its popularity and high demand among Rare Dementia Support (RDS) members, collaborative work with RDS Canada is underway to expand the programme’s reach. Whilst a preliminary pilot evaluation of The Road Less Travelled has been conducted,^42^ further evaluations assessing its effectiveness and value are required. Nevertheless, the programme has been very well-received, demonstrating its acceptability and meeting a significant need within the RDS community. NICE acknowledges the special nature of rare conditions, and apply significantly higher cost-effectiveness thresholds in these circumstances, set at a maximum of £300 000 per QALY gained.^18,43^ Although we were not able to calculate the cost per QALY of The Road Less Travelled, it is important to recognise the higher thresholds accepted by NICE when considering the value of interventions for rare diseases.

The economic cost of bereavement is challenging to estimate,^44^ however, programmes that aim to proactively prepare people to deal with grief and loss may help reduce these costs. Not only do these types of programmes help individuals prepare for future grief and loss, they also support the active process of caregiving and ‘living grief’ which many carers of people with dementia will continue to be engaged in for some time, and in some cases, for many years. Our findings add to the evidence base in this area and the costings will be used to inform a future economic modelling study of the costs and benefits of online support groups for people with rare dementia and their carers.

Due to the online mode of delivery, The Road Less Travelled was accessible to participants from a large geographical area. The BIA provides an initial estimate of costs to adopt and scale up this programme to a national extent to make the programme available to the carers of people with rare dementia in the UK. Further exploration of additional costs, such as the costs of scaling up training of facilitators to deliver the programme at this level, warrant consideration. In addition, the costs of programmes may not remain stable over time, noting that these costings reflect a pilot stage of delivery where the programme continues to develop, and on-going training was embedded within this. It is possible that programme delivery may become more efficient with less time required to prepare sessions due to increased experience. In contrast, factors such as staff turnover and the training of new staff may require additional resources, which have not yet been factored into the overall programme costs. Moreover, the very high costs associated with facilitator planning activities reported in this analysis may reflect initial set up costs during this pilot stage of intervention delivery and therefore may be expected to decrease over time and transition into training costs.

## Conclusion

To our knowledge, this is first costing analysis of a pre-death grief and loss programme for carers of people with a rare dementia. Online programmes have the potential to reduce costs and due to the rarity of this condition, it may be less cost-efficient and unfeasible to populate groups of carers of people with rare dementias in face-to-face settings. The online delivery of many programmes has received increased attention due to the Covid-19 pandemic. Our estimates indicate lower online delivery costs compared to in-person delivery. These initial assessments of costs provide a base case for future costing analyses and full economic analysis which can assess both the cost and benefits to society from supporting people with rare dementias and their carers.

## Supplemental Material

sj-docx-1-inq-10.1177_00469580251332770 – Supplemental material for The Road Less Travelled: A Micro-Costing Analysis of an Online Pre-Death Grief and Loss Programme for Carers of People with a Rare Dementia Supplemental material, sj-docx-1-inq-10.1177_00469580251332770 for The Road Less Travelled: A Micro-Costing Analysis of an Online Pre-Death Grief and Loss Programme for Carers of People with a Rare Dementia by Bethany F. Anthony, Jill Walton, Emily V. Brotherhood, Sebastian J. Crutch and Rhiannon T. Edwards in INQUIRY: The Journal of Health Care Organization, Provision, and Financing

sj-docx-2-inq-10.1177_00469580251332770 – Supplemental material for The Road Less Travelled: A Micro-Costing Analysis of an Online Pre-Death Grief and Loss Programme for Carers of People with a Rare Dementia Supplemental material, sj-docx-2-inq-10.1177_00469580251332770 for The Road Less Travelled: A Micro-Costing Analysis of an Online Pre-Death Grief and Loss Programme for Carers of People with a Rare Dementia by Bethany F. Anthony, Jill Walton, Emily V. Brotherhood, Sebastian J. Crutch and Rhiannon T. Edwards in INQUIRY: The Journal of Health Care Organization, Provision, and Financing

sj-docx-3-inq-10.1177_00469580251332770 – Supplemental material for The Road Less Travelled: A Micro-Costing Analysis of an Online Pre-Death Grief and Loss Programme for Carers of People with a Rare Dementia Supplemental material, sj-docx-3-inq-10.1177_00469580251332770 for The Road Less Travelled: A Micro-Costing Analysis of an Online Pre-Death Grief and Loss Programme for Carers of People with a Rare Dementia by Bethany F. Anthony, Jill Walton, Emily V. Brotherhood, Sebastian J. Crutch and Rhiannon T. Edwards in INQUIRY: The Journal of Health Care Organization, Provision, and Financing
