# Supplementary material for: The Road Less Travelled: A Micro-Costing Analysis of an Online Pre-Death Grief and Loss Programme for Carers of People with a Rare Dementia
Source: Inquiry. 2025 Apr 21;62:00469580251332770. doi: 10.1177/00469580251332770 (PMC12035052; doi:10.1177/00469580251332770)
Supplement: sj-docx-1-inq-10.1177_00469580251332770 – Supplemental material for The Road Less Travelled: A Micro-Costing Analysis of an Online Pre-Death Grief and Loss Programme for Carers of People with a Rare Dementia [file sj-docx-1-inq-10.1177_00469580251332770.docx]

**Supplemental Appendix A:** The Road Less Travelled programme development.

**Context:**

Rare forms of dementia affect approximately 25% of people living with dementia with many of these occurring before the age of 65.

Pre-death grief can impact individuals over many years and is a particularly complex process among those caring for people with rare dementia. The development of targeted interventions is crucial to support the unique complexities of pre-death grief in this population. Rare conditions disperse carers, limiting support and connection with others with similar experiences.

**Identification of intervention development frameworks and existing relevant interventions:**

The programme was informed by an internal literature review to identify existing pre-death grief and loss interventions for carers of people living with dementia. Existing evidence of pre-death grief interventions for carers have predominantly focused on typical forms of dementia. There is a lack of evidence for interventions for carers of people living with a rare dementia. Road Less Travelled programme’s theoretical foundation draws from established concepts from the dementia pre-death grief and loss literature (Blandin and Pepin, 2017; Doka, 2008; Moore et al., 2020; Meuser and Marwit, 2001; Noyes et al., 2010; Pearlin et al., 1990). It also drew on an evaluation of grief interventions for people living with dementia (Ott et al., 2010; Paun et al., 2015; Holland et al., 2009).

**Intervention design:**

A group based facilitated intervention delivered online to carers across the UK.

Systems-based and process-oriented logic models were developed to understand how the programme would work and to underpin the data collection required for evaluating the programme. The templates for the logic models were based on those developed by Rohwer and colleagues (2017). The system-based logic model outlined the programme, implementation, context and outcomes. This was supplemented by the process-oriented logic model which outlined the processes and causal pathways that lead from the multiple intervention components to anticipated outcomes.

The process and outcome evaluation included an assessment of whether the programme was implemented as intended, explored whether the direct and intermediate effects followed from the intervention components as anticipated, assessed the feasibility and acceptability to carers of completing outcome measures pre- and post-intervention, explored whether outcome measures are showing improvements in wellbeing from pre to post intervention, whether carers qualitatively report benefits of the programme and whether outcome measures assess the qualitative outcomes reported by carers. This informed the medium-term roll-out and subsequent evaluation across the RDS. Evaluation data included; record attendance and drop-out data, facilitator diaries (to record what they felt was working well and not working well, adaptions made to the programme), interviews with facilitators and participants post the intervention to assess their views of implementation and to test the programme logic. RDS members and a wide range of allied health professionals contributed to the design and review of the programme.

The following headings are the session topics that are covered over six sessions:

(1.) Getting to know each other (2.) Exploring and understanding grief (3.) Adapting to living loss/normalising grief (4.) Embracing identity – spouse, partner, friend, parent, child – carer?

(5.) Embracing identity – spouse, partner, friend, parent, child – carer? (6.) Programme debrief

An optional seventh session is offered (if required), to allow facilitators to cover the material adequately to allow for a more participant focused delivery.

**References**

Blandin, K. and Pepin, R. (2017). Dementia grief: a theoretical model of a unique grief experience. Dementia (London, England), 16, 67–78.

Doka, K. J. (2008). Disenfranchised grief in historical and cultural perspective. In: Stroebe, M. S., et al. (Eds.), Handbook of bereavement research and practice: Advances in theory and intervention (pp 223–240). Washington, DC, USA: American Psychological Association. <https://doi.org/10.1037/14498-011>.

Moore, K. J. et al. (2017). Experiences of end of life amongst family carers of people with advanced dementia: longitudinal cohort study with mixed methods. BMC Geriatrics, 17, 135. <https://doi.org/10.1186/s12877-017-0523-3>.

Meuser, T. M. and Marwit, S. J. (2001). A comprehensive, stage-sensitive model of grief in dementia caregiving. The Gerontologist, 41, 658–670. <https://doi.org/10.1093/geront/41.5.658>.

Noyes, B. B., Hill, R. D., Hicken, B. L., Luptak, M., Rupper, R., Dailey, N. K., & Bair, B. D. (2010). The role of grief in dementia caregiving. American Journal of Alzheimer's Disease & Other Dementias®, 25(1), 9-17.

Pearlin, L. I., Mullan, J. T., Semple, S. J., & Skaff, M. M. (1990). Caregiving and the stress process: An overview of concepts and their measures. *The gerontologist*, *30*(5), 583-594.

Ott, C. H., Kelber, S. T., & Blaylock, M. (2010). “Easing the way” for spouse caregivers of individuals with dementia: a pilot feasibility study of a grief intervention. *Research in Gerontological Nursing*, *3*(2), 89-99.

Paun, O., Farran, C. J., Fogg, L., Loukissa, D., Thomas, P. E., & Hoyem, R. (2015). A chronic grief intervention for dementia family caregivers in long-term care. Western Journal of Nursing Research, 37(1), 6-27.

Holland, J. M., Currier, J. M., & Gallagher-Thompson, D. (2009). Outcomes from the Resources for Enhancing Alzheimer's Caregiver Health (REACH) program for bereaved caregivers. Psychology and aging, 24(1), 190.

Rohwer, A., Pfadenhauer, L., Burns, J., Brereton, L., Gerhardus, A., Booth, A., ... & Rehfuess, E. (2017). Logic models help make sense of complexity in systematic reviews and health technology assessments. Journal of Clinical Epidemiology, 83, 37-47.
