# Supplementary material for: The Road Less Travelled: A Micro-Costing Analysis of an Online Pre-Death Grief and Loss Programme for Carers of People with a Rare Dementia
Source: Inquiry. 2025 Apr 21;62:00469580251332770. doi: 10.1177/00469580251332770 (PMC12035052; doi:10.1177/00469580251332770)
Supplement: sj-docx-3-inq-10.1177_00469580251332770 – Supplemental material for The Road Less Travelled: A Micro-Costing Analysis of an Online Pre-Death Grief and Loss Programme for Carers of People with a Rare Dementia [file sj-docx-3-inq-10.1177_00469580251332770.docx]

**Supplemental Appendix C:** **Facilitator cost diaries**

|  |  |  |  |
| --- | --- | --- | --- |
|  |  |  |  |
| **Questions for the individual facilitator:**  For waves 1, 2 and 3 of The Road Less Travelled programme, how many hours do you estimate you spent… |  |  |  |
|  |  |  |  |
| **Activity:** | **WAVE 1** | **WAVE 2** | **WAVE 3** |
| Meeting to co-ordinate sessions and themes with rest of team |  |  |  |
| Planning the sessions (individually and with your co-facilitator) |  |  |  |
| Finalising dates and details with co-ordinator |  |  |  |
| Writing descriptions of groups and other course material, slides, etc |  |  |  |
| Inviting or liaising with members ahead of first session |  |  |  |
| Delivering sessions |  |  |  |
| Immediate post-session catch-ups and debriefs with co-facilitators |  |  |  |
| Facilitator discussion groups |  |  |  |
| Liaising with and supporting group members one-to-one between sessions (not including standard DST work) |  |  |  |
| Other activities or costs (please specify) _____________ |  |  |  |
|  |  |  |  |
